# Long-term weight loss trajectories following participation in a randomised controlled trial of a weight management programme for men delivered through professional football clubs: a longitudinal cohort study and economic evaluation

**DOI:** 10.1186/s12966-018-0683-3

**Published:** 2018-06-28

**Authors:** Cindy M. Gray, Sally Wyke, Ruiqi Zhang, Annie S. Anderson, Sarah Barry, Nicki Boyer, Graham Brennan, Andrew Briggs, Christopher Bunn, Craig Donnachie, Eleanor Grieve, Ciaran Kohli-Lynch, Suzanne M. Lloyd, Alex McConnachie, Colin McCowan, Alice MacLean, Nanette Mutrie, Kate Hunt

**Affiliations:** 1 School of Social and Political Sciences, Institute of Health and Wellbeing, 25-29 Bute Gardens, University of Glasgow, Glasgow, G12 8RS UK; 2 Robertson Centre for Biostatistics, Institute of Health and Wellbeing, Boyd Orr Building, University Avenue, University of Glasgow, Glasgow, G12 8QQ UK; 3 Centre for Public Health Nutrition Research, Mailbox 7, Level 7, Ninewells Medical School, University of Dundee, Dundee, DD1 9SY UK; 4 Health Economics and Health Technology Assessment, Institute of Health and Wellbeing, 1 Lilybank Gardens, University of Glasgow, Glasgow, G12 8RZ UK; 5 Institute of Health and Wellbeing, 25-29 Bute Gardens, University of Glasgow, Glasgow, G12 8RS UK; 6 Institute for Social Marketing, Faculty of Health Sciences and Sport, University of Stirling, Glasgow, FK9 4LA UK; 7 Health Economics and Health Technology Assessment, Institute of Health & Wellbeing, 1 Lilybank Gardens, University of Glasgow, Glasgow, G12 8RZ UK; 8 MRC/CSO Social and Public Health Sciences Unit, University of Glasgow, 200 Renfield Street, Glasgow, G2 3QB UK; 9 Institute for Sport, Physical Education and Health Sciences, University of Edinburgh, 2.27 St Leonard’s Land, Holyrood Road, Edinburgh, EH8 8AQ UK

**Keywords:** Weight management, Long -term weight loss maintenance, Physical activity, Diet, Intervention, Men, Football, Cost-effectiveness

## Abstract

**Background:**

Obesity is a major public health concern requiring innovative interventions that support people to lose weight and keep it off long term. However, weight loss maintenance remains a challenge and is under-researched, particularly in men. The Football Fans in Training (FFIT) programme engages men in weight management through their interest in football, and encourages them to incorporate small, incremental physical activity and dietary changes into daily life to support long-term weight loss maintenance. In 2011/12, a randomised controlled trial (RCT) of FFIT demonstrated effectiveness and cost-effectiveness at 12 months. The current study aimed to investigate long-term maintenance of weight loss, behavioural outcomes and lifetime cost-effectiveness following FFIT.

**Methods:**

A longitudinal cohort study comprised 3.5-year follow-up of the 747 FFIT RCT participants. Men aged 35–65 years, BMI ≥ 28 kg/m^2^ at RCT baseline who consented to long-term follow-up (*n* = 665) were invited to participate: those in the FFIT Follow Up Intervention group (FFIT-FU-I) undertook FFIT in 2011 during the RCT; the FFIT Follow Up Comparison group (FFIT-FU-C) undertook FFIT in 2012 under routine (non-research) conditions. The primary outcome was objectively-measured weight loss (from baseline) at 3.5 years. Secondary outcomes included changes in self-reported physical activity and diet at 3.5 years. Cost-effectiveness was estimated at 3.5 years and over participants’ lifetime.

**Results:**

Of 665 men invited, 488 (73%; 65% of the 747 RCT participants) attended 3.5-year measurements. The FFIT-FU-I group sustained a mean weight loss of 2.90 kg (95% CI 1.78, 4.02; *p* < 0.001) 3.5 years after starting FFIT; 32.2% (75/233) weighed ≥5% less than baseline. The FFIT-FU-C group had lost 2.71 kg (1.65, 3.77; *p* < 0.001) at the 3.5-year measurements (2.5 years after starting FFIT); 31.8% (81/255) weighed ≥5% less than baseline. There were significant sustained improvements in self-reported physical activity and diet in both groups. The estimated incremental cost-effectiveness of FFIT was £10,700–£15,300 per QALY gained at 3.5 years, and £1790–£2200 over participants’ lifetime.

**Conclusions:**

Participation in FFIT under research and routine conditions leads to long-term weight loss and improvements in physical activity and diet. Investment in FFIT is likely to be cost-effective as part of obesity management strategies in countries where football is popular.

**Trial registration:**

ISRCTN32677491, 20 October 2011.

**Electronic supplementary material:**

The online version of this article (10.1186/s12966-018-0683-3) contains supplementary material, which is available to authorized users.

## Background

Rising levels of obesity are a major challenge to public health. The UK prevalence of overweight and obesity is higher in men (66.6% [95% UI 65.3, 68.0]) than women (57.2% [55.7, 58.6]) [1, 2]. In 2011, it was estimated that 11 million more UK adults will be obese by 2030, and that associated medical costs will increase by £1.9–2.0 billion/year [3]. Modest (5–10%) weight reductions sustained long term are associated with significant health benefits [4]. Although the behaviour change techniques and strategies that can help people achieve short-term weight loss are well described [5–7], longer term weight loss is less well researched, particularly in men [8, 9]. Weight loss following lifestyle interventions often peaks at around 6 months, followed by a gradual regain at a rate of 1 to 2 kg per year (often with larger regains in the earlier years [10]), with all weight lost regained within 3–5 years [11].

Football Fans in Training (FFIT) uses the appeal of the football club setting to attract men aged 35–65 years with BMI ≥ 28 kg/m^2^ to a 12-week weight management programme [12]. The programme is delivered free of charge by community coaching staff at professional football clubs to groups of up to 30 men (participant: coach ratio 15:1) at club stadia. Coaches are trained over 2 days in the FFIT delivery protocol. FFIT was specifically designed to work with, rather than against, prevailing conceptions of masculinity, whilst also taking account of best evidence in weight loss and behaviour change [13]. FFIT is ‘gender-sensitised’ in relation to context (the traditionally male environment of football clubs, men-only groups), content (information on the science of weight loss presented simply [‘science but not rocket science’], discussion of alcohol, ‘branding’ [e.g., use of football insignia on programme materials] and style of delivery (participative, peer-supported learning which encourages the men to interact for mutual learning and support, and positive male ‘banter’ to facilitate discussion of sensitive subjects).

Each weekly FFIT session combines advice on healthy eating and/or use of behaviour change techniques (‘classroom component’) with a coach-led group physical activity session using club facilities. The behaviour change techniques are those known to be effective in physical activity and dietary interventions (self-monitoring, goal setting, implementation intentions, feedback on behaviour) [5]. Social support both among participants and from their wider social networks [6] is also promoted. Throughout FFIT, men are encouraged to make small, incremental behavioural changes they can sustain long term, and to incorporate physical activity and healthy eating into their daily lives. During the randomised controlled trial (RCT) of FFIT, the 12-week active phase was followed by a light-touch weight maintenance phase until 12 months after the start of the programme: this included an invitation to a group reunion (at 9 months from the start of the programme) and six e-mail prompts from coaches. There was no further contact after 12 months.

In the RCT, 374 men allocated to the intervention group undertook FFIT under research conditions in autumn 2011 (during the trial, when the research team visited clubs for data collection), and 373 men allocated to the waitlist comparison group were invited to attend routine deliveries of FFIT in autumn 2012 (after the trial, when responsibility for programme delivery transferred to the Scottish Professional Football League [SPFL] Trust). The RCT demonstrated that FFIT was effective (the mean between-group weight loss at 12 months was 4.94 kg [95% CI 3.95, 5.94; *p* < 0.001], adjusted for baseline weight and club, in favour of the intervention group) and cost-effective (the incremental cost was £13,847 per QALY). Significant 12-month between-group differences in favour of the intervention group were also observed in self-reported physical activity and diet, and in other secondary outcomes [14].

This paper reports long-term weight loss trajectories of RCT participants from baseline to 3.5 years; change trajectories for RCT secondary outcomes (including self-reported physical activity and diet) from baseline to 3.5 years; and the 3.5-year and lifetime cost-effectiveness of the FFIT programme. Comparison of long-term trajectories of the intervention and comparison groups allows investigation of long-term outcomes following research and routine delivery conditions.

## Methods

### Study design and setting

This was a longitudinal cohort study, consisting of the long-term follow up of FFIT RCT participants at 13 SPFL clubs, with measurements conducted between March and September 2015, 3.5 years after RCT baseline measures. Men who consented to future research at 12-month RCT measurement sessions (665/747) were eligible to take part. As the comparison group had the opportunity to take part in the FFIT programme immediately after the 12-month measures, the long-term follow up was treated as a cohort study. The primary outcome was change in weight from baseline to 3.5 years. The protocol is available at https://www.journalslibrary.nihr.ac.uk/programmes/phr/139932/#/.

### Participant recruitment

Participants were contacted by letter from February 2015, then telephoned to arrange an appointment for the 3.5-year measurements at their club's home stadium. Data collection was undertaken by fieldstaff trained to the RCT measurement and questionnaire administration protocols. At 3.5 years, the RCT intervention and comparison groups were measured in the same stadia sessions; no attempt was made to conceal original trial group allocations, but fieldstaff were not explicitly informed of group membership. To maximise retention, multiple telephone, email and SMS contacts were made, and participants were offered measurement at home or at another convenient location if unable to attend club stadia. Those who did not take part in the full measurements could provide weight only (either objectively-measured by fieldstaff, or self-reported).

### Data collection procedures

The primary outcome, weight (kg), was recorded with electronic scales (Tanita HD 352); participants were instructed to wear light clothing, remove their shoes and empty their pockets.

Objectively-measured secondary outcomes were also assessed by fully-trained fieldstaff. Waist circumference was measured twice (three times, if the first two measurements differed by ≥5 mm); the mean of all recorded measurements was calculated. Body composition was measured using a Bodystat 1500MDD machine. Resting blood pressure was measured with a digital blood pressure monitor (Omron HEM-705CP) by a nurse. All equipment was calibrated prior to fieldwork.

Self-reported secondary outcomes were assessed through self-administered questionnaires, with fieldstaff assisting anyone with reading difficulties. Physical activity was assessed using the International Physical Activity Questionnaire (IPAQ, Short Form) [15] and scored using the IPAQ scoring protocol [16] to provide MET-min per week for self-reported total, vigorous, and moderate physical activity, and walking. Frequency of intake of various food-types was measured using an adaptation of the Dietary Instrument for Nutrition Education (DINE) [17]. Fatty food (range 8–68), fruit and vegetables (range 0.5–6.0), and sugary food (range 3–16) scores were calculated following the protocol used in the RCT [14]. Higher scores indicate higher consumption. Portion sizes of four foods important in weight gain (cheese, red meat, pasta, and chips) were assessed using eight photographs representing different portion sizes for each food [18]. Higher scores (range 1–8) represent larger portions. The total number of alcohol units consumed was assessed with a 7-day recall diary [19]. Psychological outcomes were assessed with the Rosenberg Self-Esteem (RSE) Scale [20] and the Short Form of the Positive and Negative Affect Scale (PANAS) [21]. High normalised RSE scores (range 0–3) indicate better self-esteem. Higher scores on PANAS normalised scales (range 1–5) indicate greater positive and negative affect, respectively. Health-related quality of life (HRQoL) was assessed with the 12-item Short Form Health Survey (SF-12) [22]. Higher summary scores for mental and physical health represent better HRQoL.

Participant characteristics were recorded at RCT baseline measurements in 2011 and included: age; employment status; educational attainment; socioeconomic status of postcode of residence (quintiles of Scottish Index of Multiple Deprivation [SIMD] score [23]); marital status; housing status; and ethnic origin.

### Statistical analysis

Assuming 80% of eligible participants would take part, and the standard deviation of the percentage change in weight would be 15%, we estimated the study would have 80% power to detect a change in weight of at least 2.5% in each group separately, based on a 5% two-sided significance level. All participants with available data were included in analysis. Non-response bias was investigated by comparing the baseline characteristics of participants who agreed to take part in the 3.5-year measurements with those who were not followed up using appropriate statistical tests (t-test/Mann-Whitney/chi-squared/Fisher’s exact).

To investigate long-term changes, outcomes were summarised separately by group (FFIT Follow Up Intervention “FFIT-FU-I” and FFIT Follow Up Comparison “FFIT-FU-C”), and overall. Wilcoxon signed-rank tests assessed change from baseline within groups, and Mann-Whitney tests assessed between-group differences. All outcomes were continuous. Each group was also analyzed separately within mixed effects (repeated measures) linear regression models adjusted for baseline value and measurement point (12 months and 3.5 years) as fixed effects, and for participant and club as random effects. Between-group differences in weight loss and other outcome trajectories were investigated by considering both groups together and including additional fixed effect terms for group, and the group x measurement time point interaction.

Sensitivity analyses for the primary outcome were conducted using return to baseline and last value carried forward methods to impute missing data, and using data from RCT baseline and 12-month measures as different baselines for the FFIT-FU-I and FFIT-FU-C groups, respectively, to account for the fact that the groups undertook the intervention at different times. An additional sensitivity analysis was conducted removing men who provided weight-only data at 3.5 years, including those who provided self-reported weight. Analyses were conducted using SAS Enterprise Guide (v5.1). Data are presented as mean (95% CI) or median (IQR).

### Cost-effectiveness

All cost-effectiveness analyses require a ‘no active intervention’ or ‘standard care’ control. However, because the comparison group had the opportunity to take part in the FFIT programme soon after the RCT 12-month measures, they could not be used as the control for the 3.5-year cost-effectiveness analyses. It was therefore necessary to construct hypothetical scenarios to operate as counterfactuals. We did this in two ways: first, by extrapolating RCT comparison group baseline data to take account of the fact that 11% of men in the comparison group had lost ≥5% of their body weight at the RCT 12-month measurements (i.e., before taking part in the FFIT intervention) [14]. Second, by extrapolating comparison group 12-month data (i.e., using the last observed data for the comparison group, which is likely to provide the most conservative cost-effectiveness estimate). Using these data, we modelled two possible weight trajectories: first, an average population trajectory (0.46 kg per year, the mean weight gain in men in the European Prospective Investigation into Cancer and Nutrition [EPIC] study [24]); and second, the mean annual weight gain of the FFIT-FU-I group from 12 months to 3.5 years (see Results: Primary outcome analysis). These trajectories were thought to be the most likely lower and upper weight gain boundaries. We produced six hypothetical control scenarios as follows (and see also Additional file 1, Table S1):

1. Base Case: comparison group data extrapolated from baseline, assuming that the controls put on weight from baseline to 3.5 years according to an average population trajectory [24].
2. Scenario 1: comparison group data extrapolated from baseline, assuming that the controls put on weight from baseline to 3.5 years at the same rate as the FFIT-FU-I group from 12 months to 3.5 years.
3. Scenario 2: comparison group data extrapolated from 12 months, assuming that the controls put on weight after the RCT (12 months-3.5 years) according to an average population trajectory.
4. Scenario 3: comparison group data extrapolated from 12 months, assuming that the controls put on weight after the RCT (12 months-3.5 years) at the same rate as the FFIT-FU-I group from 12 months to 3.5 years.
5. Scenario 4: comparison group data (excluding the 11% of men with ≥5% weight loss at the RCT 12-month measures [14]) extrapolated from 12 months, assuming that the controls put on weight after the RCT (12 months-3.5 years) according to an average population trajectory.
6. Scenario 5: comparison group data (excluding the 11% of men with ≥5% weight loss at the RCT 12-month measures) extrapolated from 12 months, assuming that the controls put on weight after the RCT (12 months-3.5 years) at the same rate as the FFIT-FU-I group from 12 months to 3.5 years.

The cost of providing the FFIT programme in the 13 SPFL clubs in the RCT was estimated to be £61,700, which is equivalent to £164 per FFIT participant [25]. Self-reported data on the number and type of any NHS resources used in the preceding 12-week period were collected at all time points (RCT baseline, 12 weeks and 12 months, and 3.5-year follow up) from each participant. Unit costs for visits to the GP, practice nurse or physiotherapist and any attendances at accident and emergency departments were taken from Personal Social Services Research Unit (PSSRU) costs [26, 27]. Unit costs for inpatient stays and outpatient appointments were taken from Information and Statistics Division Scotland tariffs and NHS reference costs [28]. Self-reported data on GP prescriptions of antidepressants, painkillers, asthma, pain gels/creams, anti-inflammatories and sleeping tablets (i.e., medications most likely to be affected by the intervention) were costed using unit costs from the British National Formulary [29] (see Additional file 1, Tables S2-S4). Finally, to estimate the total health resource costs associated with participation in FFIT over the entire 3.5-year period, we imputed costs at £16 per year per BMI unit increase, as estimated in the UK Counterweight Programme [30] between 12 months and 3.5 years, assuming no inflation over the period. Costs were considered from an NHS and Personal Social Service perspective (2014 GBP), and both costs and utilities discounted at 3.5% following National Institute of Health and Care Excellence (NICE) guidance [31].

We converted SF-12 scores from baseline, 12 weeks, 12 months and 3.5 years into health utility weights using the SF-6D algorithm [32]. These health utility scores were regressed against BMI and age in order to predict scores at 3.5 years in each of the hypothetical controls. A cluster variable was included in the regression, given the multiple observations per participant. Age was dropped as it was found not to be associated with utilities (see Additional file 1, Tables S5 and S6). Values were fitted for each of the six hypothetical controls by taking each individual’s BMI in each scenario as the predictor of their utility.

A longer term analysis employed the cardiovascular disease (CVD) Policy Model [33] to extrapolate 3.5-year results over participants’ lifetime. The model was updated and adapted to replace two cholesterol variables with a single BMI variable using the same dataset employed in the development of the original CVD Policy Model [34]. Weight and systolic blood pressure were assumed to be the modifiable risk factors which impact on life expectancy, quality-adjusted life years (QALYs) and costs. Systolic blood pressure (SBP) was imputed for each hypothetical control scenario informed by a systematic review which found that 10% weight loss equates to a 6.1 mmHg drop in SBP [11] (see Additional file 1, Table S7). Uncertainty around model estimates was assessed through probabilistic sensitivity analysis, and cost-effectiveness acceptability curves were produced. Uncertainty about the long-term sustainability of behavioural change was examined through a sensitivity analysis which limited the timeframe for the risk reduction impact of the intervention to 2 years beyond the 3.5-year follow up period (i.e., 5.5 years in total).

## Results

**Fig. 1:**
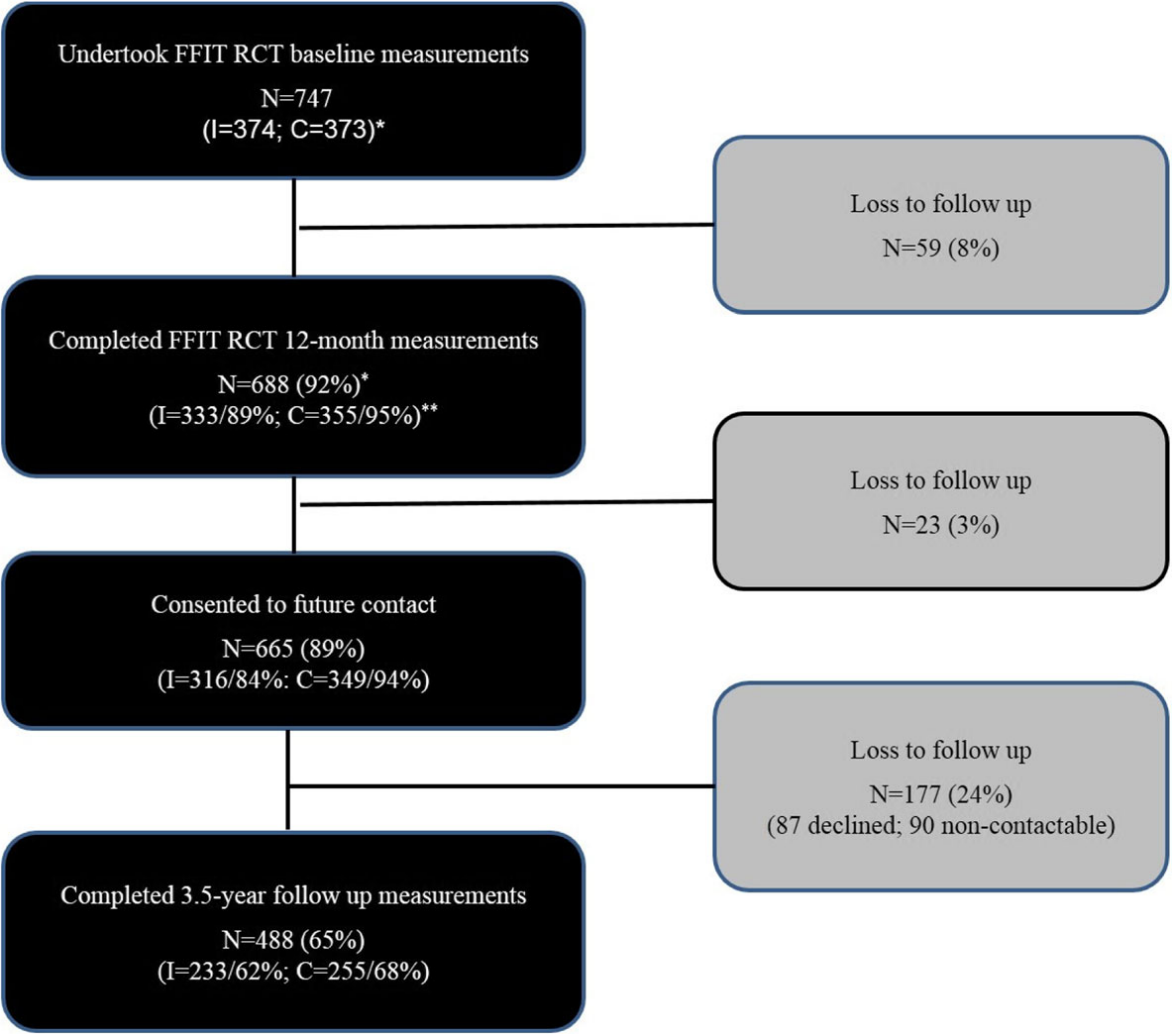
Summary of flow of participants through the FFIT RCT and FFIT Follow up Study.* the number of men enrolled in the FFIT RCT (overall and by group) is the denominator in all percentages**I = intervention group; C = comparison group

Figure 1 shows the flow of participants from RCT baseline to 3.5 years. 665/747 (89%) men consented to future follow up at RCT 12-month measurements; 87 (13%) of the 665 declined measurement at 3.5 years; a further 90 (13%) were uncontactable despite multiple attempts. Thus, 488 men took part in 3.5-year measurements (73% of those who had consented; 65% of original RCT participants). The FFIT-FU-I group comprised 62% (233/374) of men in the RCT intervention group; the FFIT-FU-C group comprised 68% (255/373) of men in the RCT comparison group. 333 men attended stadia measurement sessions, 118 completed measurements at home visits, and 37 provided weight-only data (3 weighed by fieldstaff at home visits; 34 self-reported weight).

Men who did not attend the 3.5-year measurements (“No Follow Up”) had somewhat higher RCT baseline weight (*p* < 0.001), waist circumference (*p* < 0.001), BMI (*p* < 0.001), percentage body fat (*p* = 0.002), systolic (*p* = 0.008) and diastolic blood pressure (*p* = 0.010), and were slightly younger (*p* = 0.027) and less likely to be in paid employment (*p* < 0.001) or home owners (*p* = 0.004) than those who took part in the follow up study (“FU Cohort”) (Table 1). Very similar 12-month weight losses were observed for: the No Follow Up group (3.03 kg [95% CI 1.99–4.07]) compared to the FU Cohort (2.98 kg [2.35, 3.60]); and the FFIT-FU-I group (5.49 kg [4.47, 6.51]) and FFIT-FU-C group (0.68 kg [0.03, 1.32]) compared to all men measured at 12 months in the RCT intervention group (5.56 kg [4.70, 6.43]) and comparison group (0.58 kg [0.04, 1.12]), respectively [14]. Other baseline characteristics are provided in Additional file 2 (Table S1).

**Table 1:** RCT baseline characteristics of participants in the Football Fans in Training RCT, and followed up and not followed up cohorts

	RCT Cohort (*n* = 747)	No Follow Up (*n* = 259)	FU Cohort (*n* = 488)	FFIT-FU-I (*n* = 233)	FFIT-FU-C (*n* = 255)
Objectively-measured clinical characteristics					
Weight (kg)	109.5 (17.3)	112.6 (17.2)	107.8 (17.1)	108.3 (17.9)	107.4 (16.3)
Waist (cm)	118.4 (11.7)	120.7 (11.7)	117.1 (11.6)	117.5 (12.3)	116.8 (10.8)
BMI (kg/m^2^)	35.4 (5.0)	36.3 (5.0)	34.9 (4.9)	35.0 (5.1)	34.8 (4.7)
Body fat (%)	31.7 (5.5)	32.5 (5.0)	31.2 (5.6)	31.3 (6.0)	31.2 (5.3)
*Missing*	*10*	*3*	*7*	*4*	*3*
Blood Pressure (mm/Hg)					
Systolic	140.3 (16.3)	142.5 (17.0)	139.1 (15.8)	137.5 (16.7)	140.7 (14.9)
Diastolic	88.8 (10.2)	90.2 (10.7)	88.1 (9.9)	87.4 (10.0)	88.8 (9.8)
*Missing*	*2*	*2*	*0*	*0*	*0*
Age	47.1 (8.0)	46.2 (7.8)	47.5 (8.0)	47.3 (8.2)	47.7 (7.9)
Scottish Index of Multiple deprivation (quintiles)^a^					
1 (most deprived)	131 (17.8)	45 (17.7)	86 (17.8)	40 (17.3)	46 (18.3)
2	131 (17.8)	52 (20.5)	79 (16.4)	35 (15.2)	44 (17.5)
3	122 (16.6)	42 (16.5)	80 (16.6)	43 (18.6)	37 (14.7)
4	165 (22.4)	52 (20.5)	113 (23.4)	58 (25.1)	55 (21.8)
5 (least deprived)	188 (25.5)	63 (24.8)	125 (25.9)	55 (23.8)	70 (27.8)
*Missing*	*10*	*5*	*5*	*2*	*3*
Employment Status^a^					
Paid work	626 (84.0)	210 (81.4)	416 (85.4)	201 (86.6)	215 (84.3)
Education or training	8 (1.1)	8 (3.1)	0 (0.0)	0 (0.0)	0 (0.0)
Unemployed	27 (3.6)	13 (5.0)	14 (2.9)	3 (1.3)	11 (4.3)
Not working^c^	16 (2.1)	3 (1.2)	13 (2.7)	8 (3.4)	5 (2.0)
Retired	32 (4.3)	9 (3.5)	23 (4.7)	10 (4.3)	13 (5.1)
Other	36 (4.8)	15 (5.8)	21 (4.3)	10 (4.3)	11 (4.3)
*Missing*	*2*	*1*	*1*	*1*	*0*
Housing Tenure^a^					
Owner-occupied	563 (75.4)	179 (69.1)	384 (78.7)	182 (78.1)	202 (79.2)
Other	184 (24.6)	80 (30.9)	104 (21.3)	51 (21.9)	53 (20.8)
Self-reported Physical Activity (IPAQ)^b^					
Total MET-mins/week	1188 (396, 2559)	1173 (396, 2739)	1188 (396, 2460)	1230 (396, 2460)	1155 (396, 2445)
Vigorous MET-mins/week	0 (0, 720)	0 (0, 720)	0 (0, 720)	0 (0, 720)	0 (0, 640)
Moderate MET-mins/week	0 (0, 360)	0 (0, 360)	0 (0, 360)	0 (0, 320)	0 (0, 360)
Walking MET-mins/week	446 (99, 1188)	495 (99, 1040)	396 (99, 1188)	454 (99, 1386)	396 (99, 1188)
*Missing*	*5*	*2*	*3*	*1*	*2*
Daily time spent sitting (mins)	450 (300, 600)	435 (300, 600)	465 (300, 600)	480 (300, 600)	420 (300, 600)
*Missing*	*146*	*64*	*82*	*40*	*42*
Self-reported eating and alcohol intake					
Fatty food score (DINE) (range 8–58)	23.6 (7.2)	22.9 (7.2)	23.9 (7.2)	24.1 (7.1)	23.8 (7.3)
Sugary food score (DINE) (range 3–16)	6.1 (2.8)	5.9 (2.7)	6.2 (2.9)	6.0 (2.7)	6.3 (3.0)
Fruit and vegetables score (DINE) (range 1–6)	2.3 (1.7)	2.2 (1.6)	2.3 (1.7)	2.3 (1.7)	2.3 (1.7)
Cheese portion size	4.3 (2.0)	4.2 (2.0)	4.4 (2.0)	4.4 (2.0)	4.4 (1.9)
Red meat portion size	5.6 (1.3)	5.5 (1.4)	5.7 (1.3)	5.7 (1.3)	5.7 (1.3)
Pasta portion size	5.1 (1.7)	5.0 (1.8)	5.2 (1.7)	5.3 (1.6)	5.1 (1.7)
Chips portion size	4.1 (1.8)	4.0 (1.7)	4.1 (1.8)	4.1 (1.9)	4.0 (1.7)
Total units of alcohol per week	16.7 (17.4)	16.5 (17.4)	16.9 (17.4)	15.9 (16.9)	17.8 (17.8)

Data are mean (SD), ^a^number (%) or ^b^median (IQR). ^c^Due to long-term sickness or disability. *IPAQ* international physical activity questionnaire, *MET* metabolic equivalent, *DINE* Dietary Instrument for Nutrition Education, *BMI* body-mass index.

### Primary outcome

**Fig. 2:**
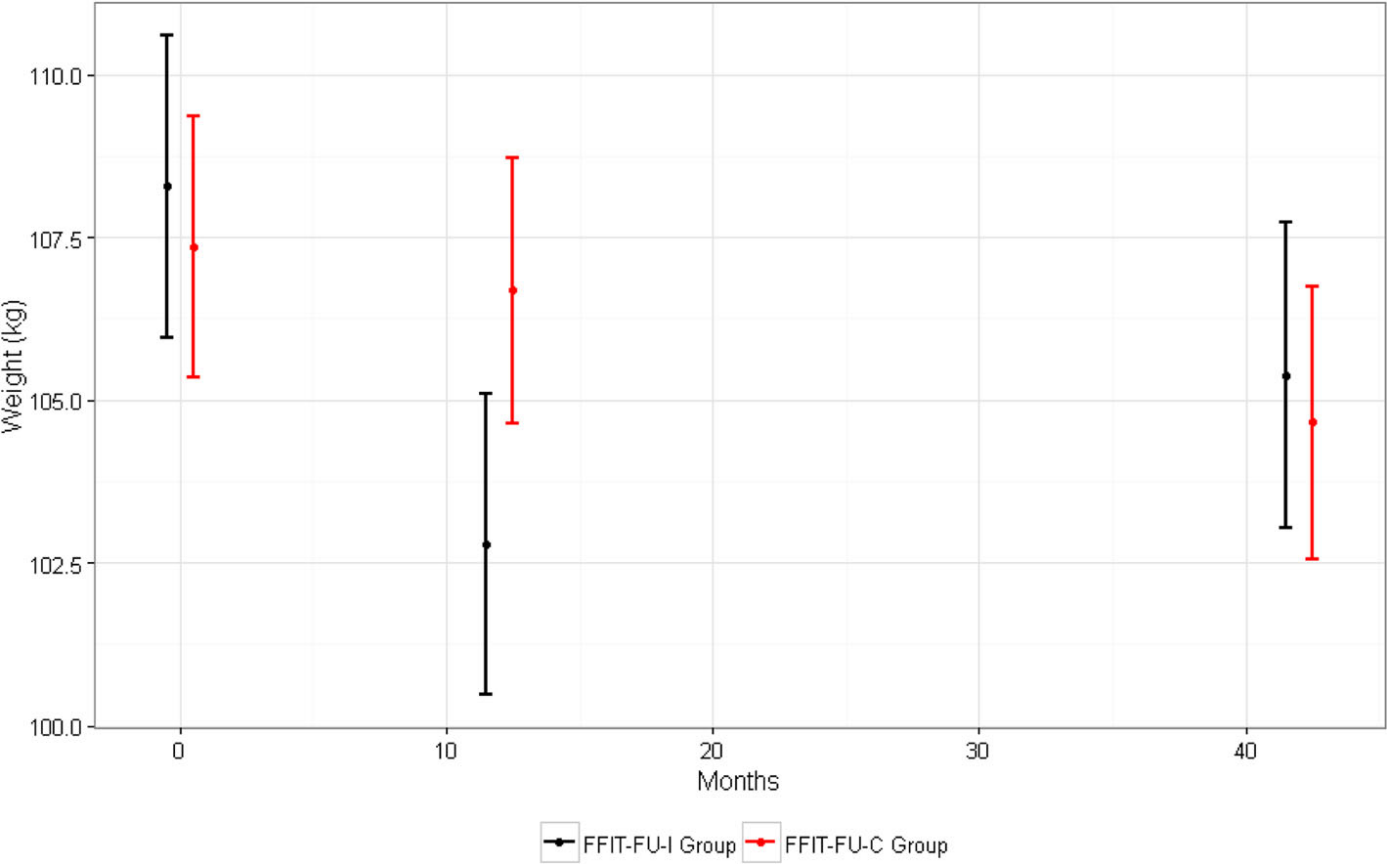
Mean weight (kg, 95% CI) in the FFIT-FU-I and FFIT-FU-C groups at RCT baseline, 12-month and 3.5-year (42-month) follow up.Note: the y-axis (weight) does not start at zero

At 3.5 years, mean weight loss from baseline was 2.90 kg (95% CI 1.78, 4.02; *p* < 0.001) or 2.52% (1.60, 3.45, *p* < 0.001) in the FFIT-FU-I group, and 2.71 kg (1.65, 3.77; *p* < 0.001) or 2.36% (1.41, 3.31; *p* < 0.001) in the FFIT-FU-C group (Table 2); and there were no between-group differences. Similar proportions of men in the FFIT-FU-I (32.2%; 75/233) and FFIT-FU-C (31.8%; 81/255) groups weighed at least 5% less than their baseline weight at 3.5 years. Figure 2 shows mean weight (95% CI) at baseline, 12 months and 3.5 years in both groups. Men in the FFIT-FU-I group gained 2.59 kg ([1.61, 3.58; *p* < 0.001], 1.04 kg per year (i.e., the annual weight gain used in the construction of the hypothetical control scenarios for the cost effectiveness analyses) between the 12-month and 3.5-year measurements; while the FFIT-FU-C group lost 2.03 kg (1.08, 2.98; *p* < 0.001) over the same period. The between-group difference in weight trajectories (− 4.62 kg [− 5.99, − 3.26; *p* < 0.001]; − 4.23% [− 5.43, − 3.02; *p* < 0.001]) is explained by men in the FFIT-FU-C group taking part in the FFIT programme immediately after the 12-month measurements. No post-programme measurements (i.e., 15 months after RCT baseline) were conducted on the FFIT-FU-C group, therefore their weight loss between 12 months and 3.5 years represents a combination of weight lost during FFIT and subsequent regain.

**Table 2:** Change from RCT baseline in objectively-measured clinical outcomes, and self-reported behavioural and psychological health outcomes at 3.5 years

	FFIT-FU-I			FFIT-FU-C			Difference	
	N	Mean (95% CI)^(a)^ or median (IQR)	*p*	N	Mean (95% CI)^(a)^ or median (IQR)	*p*	Estimate (95% CI)^(b)^	*p*
Objectively measured clinical outcomes								
Weight (kg)	233	−2.90 (−4.02, − 1.78)	< 0.001	255	− 2.71 (− 3.77, − 1.65)	< 0.001	0.19 (− 1.35, 1.73)	0.7421
Weight (%)	233	− 2.52 (− 3.45, − 1.60)	< 0.001	255	− 2.36 (− 3.31, − 1.41)	< 0.001	0.16 (− 1.17, 1.49)	0.7266
Waist (cm)	214	− 2.90 (− 3.89, − 1.91)	< 0.001	237	− 2.64 (− 3.64, − 1.65)	< 0.001	0.25 (− 1.15, 1.66)	0.706
BMI (kg/m^2^)	233	− 0.96 (− 1.31, − 0.60)	< 0.001	255	− 0.88 (− 1.22, − 0.54)	< 0.001	0.08 (− 0.42, 0.57)	0.701
Body fat (%)	162	− 1.94 (− 2.81, − 1.06)	< 0.001	165	− 1.38 (− 2.31, − 0.45)	< 0.001	0.56 (− 0.72, 1.83)	0.309
Systolic BP (mm/Hg)	214	−3.13 (−5.15, − 1.11)	0.008	235	− 4.58 (− 6.42, − 2.74)	< 0.001	−1.45 (− 4.17, 1.27)	0.186
Diastolic BP (mm/Hg)	214	−1.56 (− 2.80, − 0.32)	0.031	235	−2.95 (− 4.24, − 1.67)	< 0.001	−1.39 (− 3.18, 0.39)	0.092
Self-reported physical activity (median [IQR])								
Total MET-mins/week	213	800 (−120, 2514)	< 0.001	232	919 (−186, 2909)	< 0.001	149 (− 428, 725)	0.606
Vigorous MET-mins/week	213	0 (0, 1320)	< 0.001	232	0 (0, 1140)	< 0.001	141 (− 235, 517)	0.687
Moderate MET-mins/week	213	0 (0, 700)	< 0.001	232	0 (0, 630)	< 0.001	7 (−229, 243)	0.830
Walking MET-mins/week	213	297 (−66, 1040)	< 0.001	232	297 (−132, 1287)	< 0.001	1 (− 238, 240)	0.865
Daily time spent sitting (mins)	171	−30 (− 180, 120)	0.039	189	−30 (−180, 60)	0.001	−12 (−61, 36)	0.612
Self-reported eating and alcohol intake								
Fatty food score	214	−3.86 (− 4.83, −2.89)	< 0.001	236	− 3.16 (− 3.99, − 2.33)	< 0.001	0.70 (− 0.57, 1.97)	0.329
Sugary food score	214	−1.32 (− 1.69, − 0.95)	< 0.001	236	−1.07 (− 1.41, − 0.73)	< 0.001	0.25 (− 0.25, 0.75)	0.426
Fruit and vegetables score	214	0.50 (0.23, 0.76)	< 0.001	236	0.40 (0.14, 0.65)	0.004	−0.10 (− 0.47, 0.27)	0.560
Cheese portion size	198	−1.12 (− 1.41, − 0.83)	< 0.001	213	− 1.12 (− 1.41, − 0.83)	< 0.001	0.00 (− 0.41, 0.41)	0.939
Red meat portion size	205	−0.98 (− 1.18, − 0.77)	< 0.001	232	−0.83 (− 1.03, − 0.64)	< 0.001	0.14 (− 0.14, 0.43)	0.202
Pasta portion size	198	− 1.21 (− 1.44, − 0.98)	< 0.001	226	−1.11 (− 1.33, − 0.88)	< 0.001	0.11 (− 0.22, 0.43)	0.634
Chips portion size	183	− 1.08 (− 1.32, − 0.84)	< 0.001	217	− 0.84 (− 1.07, − 0.61)	< 0.001	0.24 (− 0.09, 0.58)	0.091
Total units of alcohol per week	207	−2.68 (− 4.52, − 0.83)	0.007	233	−4.28 (− 6.06, − 2.50)	< 0.001	− 1.61 (− 4.16, 0.95)	0.295
Self-reported psychological outcomes								
Self-Esteem	214	0.23 (0.18, 0.29)	< 0.001	237	0.25 (0.20, 0.30)	< 0.001	0.01 (− 0.06, 0.09)	0.551
Positive Affect	214	0.27 (0.17, 0.38)	< 0.001	237	0.24 (0.16, 0.32)	< 0.001	−0.04 (− 0.17, 0.09)	0.872
Negative Affect	214	−0.17 (− 0.24, − 0.11)	< 0.001	237	−0.11 (− 0.17, − 0.05)	< 0.001	0.06 (− 0.03, 0.15)	0.243
Mental HRQoL	213	1.12 (− 0.19, 2.43)	0.015	235	2.63 (1.57, 3.69)	< 0.001	1.51 (−0.17, 3.19)	0.162
Physical HRQoL	213	1.98 (0.81, 3.16)	< 0.001	235	1.09 (−0.08, 2.25)	0.022^(c)^	−0.90 (− 2.55, 0.76)	0.101

^(a)^: Within-group mean differences and 95% CIs estimated using paired t-tests

^(b)^: Between-group mean differences and 95% CIs estimated using independent t-tests

^(c)^: The confidence intervals computed assume that the physical health related quality of life variable is normally distributed and includes zero: however, the Wilcoxon signed rank sum test p-value is less than 0.05

The weight outcome sensitivity analyses showed similar results. Using the baseline carried forward method to provide data for men who did not take part in the FFIT follow up study, the FFIT-FU-I group lost 1.81 kg (1.09, 2.52; *p* < 0.001) and the FFIT-FU-C group lost 1.85 kg (1.12, 2.58; *p* < 0.001). Using the last observation carried forward method, the FFIT-FU-I group lost 3.59 kg (2.75, 4.43; *p* < 0.001) and the FFIT-FU-C group lost 1.97 kg (1.19, 2.76; *p* < 0.001). The removal of the 37/488 men who provided weight only at 3.5 years (including the 34 who provided self-reported weight) did not substantially change the 3.5-year weight results (the FFIT-FU-I group lost 3.02 kg [1.86, 4.18] and the FFIT-FU-C group lost 2.80 kg [1.70, 3.90]).

### Secondary outcomes

As Table 2 shows, both groups showed sustained improvements from baseline to 3.5 years in: self-reported physical activity (total, vigorous, moderate and walking) and daily sitting time; self-reported diet (consumption of fatty food, sugary food, fruit and vegetables, and alcohol, and portion sizes of cheese, red meat, pasta, and chips), and there were no between-group differences. There were also sustained improvements and no between-group differences in objectively-measured waist circumference, BMI, percentage body fat, and systolic and diastolic BP; and psychological indicators (self-esteem, positive and negative affect, and physical and mental HRQoL).

Comparison between 12-month and 3.5-year measurements for the FFIT-FU-I group (Table 3) shows improvements following participation in FFIT were sustained (no significant difference between 12 months and 3.5 years) for moderate physical activity, walking, sitting time, intake of fatty food, sugary food, and alcohol, and portion sizes of cheese and red meat, but not for total and vigorous physical activity, intake of fruit and vegetables, and portion sizes of pasta and chips. The same comparison for the FFIT-FU-C group allows an estimation of the impact of doing the FFIT programme after the RCT 12-month measurements (combined with any subsequent attenuation of any impact of the programme), and shows significant improvements in total and moderate physical activity, walking (but not sitting time) and all dietary outcomes except fruit and vegetable consumption (Table 3). The 12-month to 3.5-year trajectories of objectively-measured clinical outcomes and self-reported psychological outcomes are provided in Additional file 2 (Table S2).

**Table 3:** Changes in self-reported behavioural outcomes in the FFIT-FU-I and FFIT-FU-C groups between 12 months and 3.5 years

	FFIT-FU-I		FFIT-FU-C		Difference	
	Mean (95% CI)^(a)^	*p*	Mean (95% CI)^(a)^	*p*	Estimate (95% CI)^(b)^	*p*
Self-reported physical activity						
Total MET-mins/week	− 439 (− 871, − 8)	0.046	668 (292, 1044)	< 0.001	1096 (526, 1666)	< 0.001
Vigorous MET-mins/week	− 542 (− 824, − 261)	< 0.001	219 (− 58, 496)	0.120	760 (366, 1155)	< 0.001
Moderate MET-mins/week	45 (−118, 208)	0.586	210 (46, 374)	0.012	161 (−70, 393)	0.172
Walking MET-mins/week	55 (−115, 226)	0.523	232 (67, 398)	0.006	176 (−62, 413)	0.147
Daily time spent sitting (mins)	24 (−7, 56)	0.133	−16 (−43, 12)	0.257	−40 (−82, 1)	0.057
Self-reported eating and alcohol intake						
Fatty food score	0.71 (−0.10, 1.53)	0.086	−1.15 (− 1.90, − 0.40)	0.003	−1.88 (− 2.98, − 0.77)	< 0.001
Sugary food score	−0.02 (− 0.32, 0.29)	0.917	−0.54 (− 0.86, − 0.22)	0.001	−0.54 (− 0.98, − 0.09)	0.018
Fruit and vegetables score	−0.42 (− 0.67, − 0.17)	0.001	0.19 (− 0.06, 0.43)	0.130	0.60 (0.25, 0.95)	< 0.001
Cheese portion size	0.22 (− 0.04, 0.48)	0.090	− 0.39 (− 0.63, − 0.15)	0.002	−0.61 (− 0.96, − 0.26)	< 0.001
Red meat portion size	0.09 (− 0.11, 0.29)	0.374	−0.31 (− 0.51, − 0.12)	0.002	−0.40 (− 0.68, − 0.12)	0.005
Pasta portion size	0.32 (0.12, 0.52)	0.002	−0.43 (− 0.64, − 0.23)	< 0.001	−0.76 (− 1.05, − 0.47)	< 0.001
Chips portion size	0.34 (0.16, 0.53)	< 0.001	− 0.26 (− 0.45, − 0.06)	0.009	−0.61 (− 0.88, − 0.34)	< 0.001
Total units of alcohol per week	0.69 (− 0.95, 2.33)	0.408	− 1.68 (− 3.31, − 0.04)	0.045	−2.42 (− 4.74, − 0.10)	0.041

^(a)^: Within-group means and 95% CIs estimated using repeated measures models adjusted for baseline and measurement time point (baseline, 12 months and 3.5 years) as fixed effects, and for participant and club as random effects

^(b)^: Between-group mean differences estimated using repeated measures models adjusted for baseline, group, measurement time point (baseline, 12 months and 3.5 years), and the group × measurement time point interaction as fixed effects, and for participant and club as random effects

### Economic evaluation

At 3.5 years, the total costs associated with the FFIT intervention were estimated as £571,000 (95% CI £401,000, £740,000); a mean cost of £2450 per participant (which included the cost of the programme [£164 per participant], as well as self-reported use of health care resources; i.e., visits to the GP, practice nurse or physiotherapist, attendances at accident and emergency departments, hospital inpatient stays and outpatient appointments, and GP prescriptions for antidepressants, painkillers, asthma, pain gels/creams, anti-inflammatories and sleeping tablets). Estimates of the total costs associated with the six hypothetical ‘no active intervention’ scenarios ranged from £521,000 (£410,000, £632,000) to £697,000 (£480,000, £914,000); a mean cost of between £1640 and £1870 per participant.

These figures demonstrate that FFIT was more expensive than ‘no active intervention’ over 3.5 years, with an additional discounted cost of £532–£740 per participant. The intervention is also more effective, with an average gain in quality-adjusted life years (QALYs) of 0.046–0.051 across the hypothetical scenarios. This results in an incremental cost-effectiveness of £10,700–£15,300 per QALY gained.

In the lifetime analysis, FFIT was associated with an incremental cost of £1450–£1680 per participant, and an average gain in QALYs of 0.679–0.821 across the hypothetical scenarios. This results in an incremental cost-effectiveness of £1790–£2200 per QALY gained within participants’ lifetimes (details are provided in Additional file 2, Table S3).

The sensitivity analysis indicated that FFIT remained cost-effective when the beneficial impact of the intervention was limited to 5.5 years. Specifically, although FFIT remained more expensive than ‘no active intervention’, the average additional cost was reduced to £1025 (95% CI £85, £1220) per participant. The sensitivity analysis also indicated that FFIT remained more effective, but to a lesser extent, with an average increase of 0.639 (0.595, 0.693) QALYs. The cost-effectiveness acceptability curves for the lifetime and 5.5-year sensitivity analyses (see Additional file 2, Figure S1) demonstrate that FFIT starts to become a cost-effective option when a decision-maker is prepared to pay around £2000 per QALY.

## Discussion

Participation in the FFIT intervention is associated with sustained long-term weight loss. Men in the trial intervention group who undertook FFIT immediately after the randomised controlled trial baseline measures (FFIT-FU-I group) weighed on average 2.90 kg less at 3.5 years than they did at baseline; and almost a third had sustained a clinically important weight loss of at least 5% 3.5 years after starting the programme. They also showed sustained improvements in: self-reported physical activity; intake of fatty foods, sugary foods, fruit and vegetables, and alcohol; portion sizes; waist circumference; percentage body fat; BMI; blood pressure; self-esteem; positive and negative affect; and physical and mental HRQoL. The programme was highly cost-effective, both over 3.5 years and over participants’ lifetime.

The long-term weight, physical activity, dietary and psychological outcomes of the FFIT-FU-C group were very similar to the FFIT-FU-I group at the 3.5-year measures (i.e., 2.5 years after the RCT comparison group had the opportunity to undertake the FFIT programme). This suggests that FFIT can be successfully delivered under routine (non-research) conditions, and that long-term outcomes from ongoing routine deliveries funded by the Scottish Government and overseen by the SPFL Trust should be similar to those obtained by men who took part in FFIT under research conditions during the RCT. The programme has now been delivered to around 4500 men in 33 Scottish professional football clubs, and to men at seven clubs in England and 12 in Germany.

The long-term weight loss in FFIT is comparable to that reported in a recent men-only weight loss maintenance trial, where 92 men (44% of the original cohort of 209 men) who lost at least 4 kg in an initial 3-month weight loss programme (mean weight loss 7.3 kg) were randomized either to take part in a 6-month weight loss maintenance programme, which comprised written materials, and SMS and video email messages (WLM), or receive no additional intervention (WL-only) [35]. Three years after completing the original weight loss programme, the WLM and WL-only groups had maintained 51 and 59% of their initial weight loss, respectively. By comparison, 3.5 years after starting the FFIT programme, the FFIT-FU-I group had maintained on average 53% of their 12-month weight loss across the whole cohort (i.e., all men were included regardless of their initial weight loss). Long-term weight loss following FFIT is lower than that reported by Borg et al. [36], where 90 men took part in a 2-month programme involving a very low energy diet, followed by an active 6-month weight maintenance phase. At 31 months, mean weight loss was 4.0–6.1 kg. However, this intervention was far more intensive than FFIT (weekly small group meetings for 8 months, rather than weekly group sessions over 12 weeks for FFIT), and the numbers taking part and followed up were much lower (only 68 men provided outcome data at 31 months).

Despite the average annual weight regain (1.04 kg per year) in the FFIT-FU-I group being more than estimates of average annual weight gain in the general population (around 0.46 kg per year) [24], it compares favourably with patterns of regain following participation in other weight loss interventions. These typically show a regain of 1 to 2 kg per year post-programme [10] (often around 30–35% of lost weight in the first year [37]), with all weight lost regained within 3–5 years [11].

Although there were decreases in self-reported total and vigorous physical activity between 12 months and 3.5 years in the FFIT-FU-I group, levels of walking and other moderate physical activity remained stable. Long-term follow ups of physical activity interventions are rare [38], therefore the current study provides important evidence of how men’s initial enthusiasm for walking during FFIT [39] has successfully translated into an ongoing behaviour. In relation to diet, the FFIT-FU-I group appeared to be successful in sustaining improvements in consumption of fatty foods, sugary foods, and alcohol, and in reducing portion sizes of cheese and red meat from 12 months to 3.5 years. In the post-programme and 12-month focus group discussions conducted during the RCT, information on portion sizes and food choices emerged as a highly valued part of the programme [25].

Our economic evaluation demonstrates that when a decision maker is willing to pay £20,000 or £30,000 per QALY (the standard UK cost-effectiveness thresholds accepted by NICE [31]), there is no uncertainty that FFIT is cost-effective assuming that the benefit is sustained across the lifetime. The results of the analysis in which the beneficial impact of the intervention was limited to 5.5 years also indicates that FFIT remains cost-effective. This finding is consistent with recent NICE economic modelling which indicates that interventions for moderately and morbidly obese groups achieving at least 1 kg weight loss are cost-effective as long as weight is not regained within 3 to 5 years [40].

### Limitations

The FFIT follow up study has a number of strengths, and some limitations. One strength is that our intensive retention strategies allowed 3.5-year follow up of 73% (488/665) of men who had consented at the RCT 12-month measurements to future contact (65% of the 747 men in the full RCT population). Although a little older and less likely to be in paid employment or home owners than non-participants, participants in this follow up study were broadly representative of the full RCT population in terms of their within-trial weight loss trajectories; and sensitivity analyses conducted to account for loss to follow up, revealed a similar pattern of results as the main weight outcome analyses. As men in the RCT comparison group took part in the FFIT programme after the end of the RCT, we were unable to collect any data on their 12-month post-programme outcomes. This means we lack important information to plot their long-term weight trajectories (hence for this group we are unable to disaggregate weight loss over the course of the 12-week programme and any subsequent regain). Nevertheless, the fact that this group undertook FFIT under routine (non-research) conditions means that we have valuable information on the long-term outcomes of men who take part in FFIT under routine conditions, and provides ecological validity to our findings.

Physical activity, diet and alcohol consumption were assessed through self-report. Although more objective measurement (e.g., accelerometry, interviewer-administered recall) might be considered desirable, this would have been logistically difficult and prohibitively expensive. As these were secondary outcomes in the original RCT, a pragmatic decision was taken that self-report would be adequate to provide an estimate of change over time in these important behaviours, recognizing the potential for response bias (e.g., inaccurate recall, social desirability) [41]. In addition, no adjustments were made for multiple statistical comparisons. *P*-values less than 0.05 were taken as suggestive of an association, with smaller *p*-values giving stronger evidence for true associations. However, it is possible that some significant results may be due to chance.

Finally, the main limitation for the economic evaluation was the lack of a ‘no active intervention’ group at 3.5 years. We addressed this by undertaking robust and multiple sensitivity analyses by modelling six hypothetical control scenarios [34].

## Conclusion

Rising levels of obesity and associated health risks demand innovative evidence-based interventions to help people lose weight and maintain this over the long term. The evidence presented shows that FFIT was effective in helping men achieve significant improvements in weight, physical activity, and dietary outcomes for up to 3.5 years, and was well within the threshold range of £20,000–£30,000 per QALY that NICE considers cost-effective [26]. The finding that similar improvements were achieved by men taking part in routine, non-research programme deliveries suggests that investment in FFIT is likely to be cost-effective as an international obesity management strategy in any country where football has a high profile and in which obesity is a problem in men.

## Additional files

Additional file 1: Economic evaluation supplementary tables. (DOCX 57 kb)

Additional file 2: Additional follow up study results tables (baseline characteristics, clinical and psychological outcomes, and lifetime economic evaluation). (DOCX 103 kb)
