# Supplementary material for: Long-term weight loss trajectories following participation in a randomised controlled trial of a weight management programme for men delivered through professional football clubs: a longitudinal cohort study and economic evaluation
Source: Int J Behav Nutr Phys Act. 2018 Jun 28;15:60. doi: 10.1186/s12966-018-0683-3 (PMC6022303; doi:10.1186/s12966-018-0683-3)
Supplement: Supplementary file 1 — Economic evaluation supplementary tables. (DOCX 57 kb) [file 12966_2018_683_MOESM1_ESM.docx]

# **Additional File 1**

Table 1. Extrapolated weight, weight trajectories and BMI for the hypothetical control scenarios in the FFIT Follow Up Study

|  | Baseline | | | | 12 months | | | | 3.5 years | | | | | | | |
| --- | --- | --- | --- | --- | --- | --- | --- | --- | --- | --- | --- | --- | --- | --- | --- | --- |
|  | FFIT-FU-I | | Hypothetical control scenarios* | | FFIT-FU-I | | Hypothetical control scenarios* | | FFIT-FU-I | | Hypothetical control scenarios* | | FFIT-FU-I | Hypothetical control scenarios* | |  |
|  | Mean weight (kg) | Mean BMI (kg/m^2^) | Mean weight (kg) | Mean BMI (kg/m^2^) | Mean weight (kg) | Mean BMI (kg/m^2^) | Mean weight (kg) | Mean BMI (kg/m^2^) | Mean weight (kg) | Mean BMI (kg/m^2^) | Mean weight (kg) | Mean BMI (kg/m^2^) | Weight gain trajectory (kg) | | Extrapolat. weight gain trajectory (kg) |  |
| Base Case | 108.28 | 35.03 | 108.55 | 35.19 | 102.79 | 33.26 | 108.12 | 35.02 | 105.38 | 34.08 | 110.16 | 35.72 | 1.04 | | 0.46 |  |
| SA1 | 108.28 | 35.03 | 108.55 | 35.19 | 102.79 | 33.26 | 108.12 | 35.02 | 105.38 | 34.08 | 112.05 | 36.33 | 1.04 | | 1.00 |  |
| SA2 | 108.28 | 35.03 | 108.55 | 35.19 | 102.79 | 33.26 | 108.12 | 35.02 | 105.38 | 34.08 | 109.27 | 35.39 | 1.04 | | 0.46 |  |
| SA3 | 108.28 | 35.03 | 108.55 | 35.19 | 102.79 | 33.26 | 108.12 | 35.02 | 105.38 | 34.08 | 110.62 | 35.83 | 1.04 | | 1.00 |  |
| SA4 | 108.28 | 35.03 | 108.46 | 35.14 | 102.79 | 33.26 | 109.30 | 35.38 | 105.38 | 34.08 | 110.45 | 35.75 | 1.04 | | 0.46 |  |
| SA5 | 108.28 | 35.03 | 108.46 | 35.14 | 102.79 | 33.26 | 109.30 | 35.38 | 105.38 | 34.08 | 111.80 | 36.19 | 1.04 | | 1.00 |  |

*3 men in the original FFIT comparison group had died by the 3.5-year follow up and were excluded from construction of the hypothetical control scenarios. SA = Scenario. Extrapolat. = extrapolated

Table 2. Unit costs of NHS resources from Personal Social Services Research Unit (PSSRU) 2011/12^1^ and 2014/15^2^

| **Type of health care visit** | **Unit cost (£)**  **2011/12** | **Unit cost (£)**  **2014/15** | **Notes** |
| --- | --- | --- | --- |
| GP visit | 43.00 | 44.00 | Per patient contact in surgery (assumed 11.7 minutes). Includes direct care staff costs. |
| Practice nurse visit | 13.25 | 14.00 | Per patient contact in surgery (assumed 15 minutes). |
| Physiotherapist | 16.50 | 18.00 | Per patient contact in community (assumed 30 minutes). No equivalent listing. Assumed band 5 of scientific and professional staff. |
| Accident and Emergency visit | 41.00 | 44.00 | In PSSRU 2011/12, used walk-in service, not admitted. As no equivalent A&E listing in PSSRU 2014/15, used £44 'hear, treat and refer' ambulance services as approximates to previous costs. |

**Table 3. Health resource use costs based on 12-week recall at 3.5 yrs (in £) (totals and sub-totals rounded to 3 significant figures)**

| **Cost category** | **FFIT-FU-I**  **N=233** | **FFIT-FU-C**  **N = 255** | **Total** | **95% CI** |
| --- | --- | --- | --- | --- |
| **Primary care** |  |  |  |  |
| GP | 6688 | 8272 | 14960 |  |
| Nurse | 1596 | 1288 | 2884 |  |
| Physio | 864 | 972 | 1836 |  |
| Emergency | 792 | 836 | 1628 |  |
| *Sub-total primary care* | *9940* | *11370* | *21300* | *18000, 24600* |
| **Medications** |  |  |  |  |
| Painkillers | 283 | 329 | 611 |  |
| Anti-inflammatories | 149 | 149 | 297 |  |
| Gels/creams | 96 | 100 | 196 |  |
| Inhalers | 74 | 129 | 204 |  |
| Sleeping pills | 6 | 10 | 16 |  |
| Anti-depressants | 40 | 42 | 82 |  |
| *Sub-total medications* | *648* | *758* | *1410* | *1190, 1620* |
| **Secondary care** |  |  |  |  |
| Outpatient | 7782 | 9970 | 17752 |  |
| Inpatient | 22156 | 13957 | 36113 |  |
| Other | 5311 | 8202 | 13513 |  |
| *Sub-total Secondary care* | *35200* | *32100* | *67400* | *28400, 106000* |
| **TOTAL** | **45800** | **44300** | **90100** | **50000, 130000** |

Table 4. Unit costs of prescribed medications from the British National Formulary^3^

| **Kind of medication** | **Unit cost (£) 2011/2** | **Unit cost (£) 2015** | **Drug costed** |
| --- | --- | --- | --- |
| Asthma | 2.19 | 2.19 | Beconase |
| Antidepressants | 0.96 | 1.02 | Citalopram: 20 mg, 28-tablet pack |
| Painkillers | 4.70 | 3.87 | Cocodamol: 8 mg, 100-tablet pack |
| Anti-inflammatories | 3.23 | 3.23 | Diclofenac potassium: 25 mg, 28-tablet pack |
| Sleeping tablets | 0.86 | 1.06 | Diazepam: 5 mg, 28-tablet pack |
| Gels/creams | 3.89 | 4.00 | Ibuprofen: maximum strength 10% w/w, 100g pack. (50g pack no longer listed) |

Table 5. Regression of SF12 utility scores, BMI and age, adjusted for participant-level clustering (rounded to 3 significant figures)

| Risk factor | Coef. | Std. Err. | t | P>t | 95%CI | |
| --- | --- | --- | --- | --- | --- | --- |
| BMI | -0.00391 | 0.000748 | -5.23 | 0.000 | -0.00537 | -0.00244 |
| Age | 0.00092 | 0.000471 | 1.95 | 0.051 | -4.95e-06 | 0.00184 |
| Constant | 0.884094 | 0.0377 | 23.5 | 0.000 | 0.810 | 0.958 |

Table 6. Regression of SF12 utility scores and BMI, adjusted for participant-level clustering (rounded to 3 significant figures)

| Risk factor | Coef. | Std. Err. | t | P>t | 95%CI | |
| --- | --- | --- | --- | --- | --- | --- |
| BMI | -0.00412 | 0.000731 | -5.64 | 0.000 | --0.00556 | -0.00269 |
| Constant | 0.936 | 0.0254 | 36.8 | 0.000 | 0.886 | 0.986 |

Table 7. Mean systolic blood pressure (mmHg) from FFIT RCT and FFIT Follow Up for the FFIT-FU-I group and with imputed systolic blood pressure for the hypothetical control scenarios

|  | Baseline | | 12 months | | 3.5 year | |
| --- | --- | --- | --- | --- | --- | --- |
|  | FFIT-FU-I | Hypoth. SAs | FFIT-FU-I | Hypoth. SAs | FFIT-FU-I | Hypoth. SAs |
| FFIT RCT | 137.45 | 141.20 | 129.70 | 134.47 | 134.68 | n/a |
| Base Case | 137.45 | 141.20 | 129.70 | 134.47 | 134.68 | 142.07 |
| SA1 | 137.45 | 141.20 | 129.70 | 134.47 | 134.68 | 143.16 |
| SA2 | 137.45 | 141.20 | 129.70 | 134.47 | 134.68 | 135.17 |
| SA3 | 137.45 | 141.20 | 129.70 | 134.47 | 134.68 | 135.95 |
| SA4 | 137.45 | 141.41 | 129.70 | 135.49 | 134.68 | 136.19 |
| SA5 | 137.45 | 141.41 | 129.70 | 135.49 | 134.68 | 136.96 |

SA = Scenario. Hypoth. SAs = Hypothetical control scenarios

**References**

1.Personal Social Services Research Unit. *Unit Costs of Health and Social Care 2011.* URL: www.pssru.ac.uk/project-pages/unit-costs/2011/index.php (accessed 16 November 2016).

2.Personal Social Services Research Unit. *Unit Cost of Health and Social Care 2015.* URL: <http://www.pssru.ac.uk/project-pages/unit-costs/2015/index.php> (accessed 16th November 2016).

3.British National Formulary. URL: www.bnf.org/bnf/index.htm (accessed 16 November 2016).

4.Scottish Government. *Scottish Index of Multiple Deprivation.* URL: <http://www.scotland.gov.uk/Topics/Statistics/SIMD/> (accessed 17 November 2016).
