# Supplementary material for: Long-term weight loss trajectories following participation in a randomised controlled trial of a weight management programme for men delivered through professional football clubs: a longitudinal cohort study and economic evaluation
Source: Int J Behav Nutr Phys Act. 2018 Jun 28;15:60. doi: 10.1186/s12966-018-0683-3 (PMC6022303; doi:10.1186/s12966-018-0683-3)
Supplement: Supplementary file 2 — Additional follow up study results tables (baseline characteristics, clinical and psychological outcomes, and lifetime economic evaluation). (DOCX 103 kb) [file 12966_2018_683_MOESM2_ESM.docx]

# Additional File 2

| **Table 1. RCT baseline characteristics of participants in the Football Fans in Training RCT, and Followed up and not followed up cohorts** | | | | | |
| --- | --- | --- | --- | --- | --- |
|  | **RCT Cohort**  **(n=747)** | **No Follow-up (n=259)** | **FU Cohort  (n=488)** | **FFIT-FU-I**  **(n=233)** | **FFIT-FU-C**  **(n=255)** |
| **Ethnic origin** |  |  |  |  |  |
| White (British, Scottish, Irish or other) | 735 (99·1) | 256 (99·2) | 479 (99·0) | 228 (98·7) | 251 (99·2) |
| Other | 7 (0·9) | 2 (0·8) | 5 (1·0) | 3 (1·3) | 2 (0·8) |
| *Missing* | *5* | *1* | *4* | *2* | *2* |
| **Education** |  |  |  |  |  |
| No qualifications | 71 (9·5) | 32 (12·4) | 39 (8·0) | 17 (7·3) | 22 (8·6) |
| Standard grades or Highers* | 241 (32·3) | 83 (32·0) | 158 (32·4) | 73 (31·3) | 85 (33·3) |
| Vocational or HNC or HND | 240 (32·1) | 82 (31·7) | 158 (32·4) | 84 (36·1) | 74 (29·0) |
| University education | 156 (20·9) | 53 (20·5) | 103 (21·1) | 48 (20·6) | 55 (21·6) |
| Other | 39 (5·2) | 9 (3·5) | 30 (6·1) | 11 (4·7) | 19 (7·5) |
| **Marital Status** |  |  |  |  |  |
| Married | 518 (69·3) | 181 (69·9) | 337 (69·1) | 149 (63·9) | 188 (73·7) |
| Living with partner | 95 (12·7) | 31 (12·0) | 64 (13·1) | 39 (16·7) | 25 (9·8) |
| Other (single, divorced or widowed) | 134 (17·9) | 47 (18·1) | 87 (17·8) | 45 (19·3) | 42 (16·5) |
| **Self-reported physical activity (IPAQ)** | | | | |  |
| Total MET-mins/week | 1188 (396, 2559) | 1173 (396, 2739) | 1188 (396, 2460) | 1230 (396, 2460) | 1155 (396, 2445) |
| Vigorous MET-mins/week | 0 (0, 720) | 0 (0, 720) | 0 (0, 720) | 0 (0, 720) | 0 (0, 640) |
| Moderate MET-mins/week | 0 (0, 360) | 0 (0, 360) | 0 (0, 360) | 0 (0, 320) | 0 (0, 360) |
| Walking MET-mins/week | 446 (99, 1188) | 495 (99, 1040) | 396 (99, 1188) | 454 (99, 1386) | 396 (99, 1188) |
| *Missing* | *5* | *2* | *3* | *1* | *2* |
| Daily time spent sitting (mins) | 450 (300, 600) | 435 (300, 600) | 465 (300, 600) | 480 (300, 600) | 420 (300, 600) |
| *Missing* | *146* | *64* | *82* | *40* | *42* |
| **Self-reported eating and alcohol intake** | | | | | |
| DINE-based measures | | | | |  |
| Fatty food score | 23·6 (7·2) | 22·9 (7·2) | 23·9 (7·2) | 24·1 (7·1) | 23·8 (7·3) |
| Sugary food score | 6·1 (2·8) | 5·9 (2·7) | 6·2 (2·9) | 6·0 (2·7) | 6·3 (3·0) |
| Fruit and vegetable score | 2·3 (1·7) | 2·2 (1·6) | 2·3 (1·7) | 2·3 (1·7) | 2·3 (1·7) |
| Cheese portion size  *Missing* | 4·3 (2·0)  *39* | 4·2 (2·0)  *14* | 4·4 (2·0)  *25* | 4·4 (2·0)  *9* | 4·4 (1·9)  *6* |
| Red meat portion size  *Missing* | 5·6 (1·3)  *9* | 5·5 (1·4)  *3* | 5·7 (1·3)  *6* | 5·7 (1·3)  5 | 5·7 (1·3) *1* |
| Pasta portion size  *Missing* | 5·1 (1·7)  *21* | 5·0 (1·8)  *5* | 5·2 (1·7)  *16* | 5·3 (1·6) *11* | 5·1 (1·7)  *5* |
| Chips portion size  *Missing* | 4·1 (1·8)  *35* | 4·0 (1·7)  *10* | 4·1 (1·8)  *25* | 4·1 (1·9)  *17* | 4·0 (1·7)  *8* |
| Total units alcohol per week | 16·7(17·4) | 16·5 (17·4) | 16·9 (17·4) | 15·9 (16·9) | 17·8 (17·8) |
| **Self-reported psychological health and quality of life** | | | | |  |
| Self-esteem (Rosenberg)† | 1·9 (0·5) | 1·9 (0·5) | 1·9 (0·5) | 1·9 (0·5) | 2·0 (0·5) |
| Positive affect (PANAS)‡ | 3·2 (0·7) | 3·2 (0·7) | 3·2 (0·7) | 3·3 (0·7) | 3·2 (0·6) |
| Negative affect (PANAS)‡ | 1·7 (0·6) | 1·7 (0·6) | 1·7 (0·6) | 1·7 (0·6) | 1·6 (0·6) |
| Mental HRQoL (Short Form 12) | 48·6 (9·7) | 48·5 (9·4) | 48·6 (9·8) | 48·9 (10·1) | 48·4 (9·5) |
| *Missing* | *1* | *0* | *1* | *1* | *0* |
| Physical HRQoL (Short Form 12) | 47·4 (7·7) | 46·6 (7·5) | 47·7 (7·8) | 47·3 (7·9) | 48·2 (7·6) |
| *Missing* | *1* | *0* | *1* | *1* | *0* |
| Data are number (%), mean (SD) or median (IQR). HNC=Higher National Certificate. HND=Higher National Diploma. IPAQ=international physical activity questionnaire. MET=metabolic equivalent. DINE=dietary instrument for nutritional education. PANAS=positive and negative affect scale. HRQoL=health-related quality of life. *Scottish school-based qualifications taken at ages 15–16 years and 17–18 years. †Normalised Rosenberg score, range 0–3. ‡Normalised PANAS score, range 1–5. | | | | | |

| **Table 2. Changes in objectively-measured clinical and self-reported psychological outcomes in the FFIT-FU-I and FFIT-FU-C groups between 12 months and 3·5 years** | | | | | | | |  |  |
| --- | --- | --- | --- | --- | --- | --- | --- | --- | --- |
|  | **FFIT-FU-I** | | **FFIT-FU-C** | | **Difference** | | | |  |
|  | **Mean^(a)^**  **(95% CI)** | **p** | **Mean^(a)^**  **(95% CI)** | **p** | | **Estimate^(b)^**  **(95% CI)** | **p** | | |
| **Objectively-measured clinical outcomes** | | | | | | | |  |  |
| Waist (cm) | 4·41  (3·46, 5·36) | <0·001 | -0·83  (-1·79, 0·12) | 0·086 | | -5·26  (-6·61,-3·91) | <0·001 | | |
| BMI (kg/m^2^) | 0·82  (0·50, 1·13) | <0·001 | -0·66  (-0·96, -0·35) | <0·001 | | -1·47  (-1·91,-1·04) | <0·001 | | |
| Body fat (%) | 0·40  (-0·44, 1·24) | 0·346 | -1·40  (-2·30, -0·50) | 0·002 | | -1·84  (-3·09,-0·59) | 0·004 | | |
| Systolic BP (mm/Hg) | 4·62  (2·98, 6·27) | <0·001 | 1·38  (-0·25, 3·01) | 0·096 | | -3·20  (-5·51,-0·88) | 0·007 | | |
| Diastolic BP (mm/Hg) | 3·05  (1·85, 4·26) | <0·001 | 0·48  (-0·65, 1·60) | 0·406 | | -2·55  (-4·19,-0·90) | 0·002 | | |
| **Self-reported psychological outcomes** | | | | | | | |  |  |
| Self-Esteem | -0·01  (-0·06, 0·03) | 0·544 | 0·12  (0·07, 0·16) | <0·001 | | 0·13  (0·07, 0·20) | <0·001 | | |
| Positive Affect | -0·06  (-0·14, 0·02) | 0·148 | 0·17  (0·10, 0·25) | <0·001 | | 0·23  (0·12, 0·34) | <0·001 | | |
| Negative Affect | 0·04  (-0·03, 0·10) | 0·273 | -0·02  (-0·07, 0·03) | 0·427 | | -0·06  (-0·14, 0·02) | 0·149 | | |
| Mental HRQoL | -1·20  (-2·36, 0·04) | 0·043 | 1·02  (0·06, 1·99) | 0·038 | | 2·22  (0·73, 3·72) | 0·004 | | |
| Physical HRQoL | -0·07  (-1·19, 1·04) | 0·897 | 0·81  (-0·23, 1·85) | 0·124 | | 0·89  (-0·63, 2·41) | 0·249 | | |
| ^(a)^: Within-group means and 95% CIs estimated using repeated measures models adjusted for baseline measure and measurement time point (baseline, 12 months and 3·5 years) as fixed effects, and for participant and club as random effects.  ^(b)^: Between-group mean differences estimated using repeated measures models adjusted for baseline measure, group, measurement time point (baseline, 12 months and 3·5 years), and the group × measurement time point interaction as fixed effects, and for participant and club as random effects. | | | | | | | |  |  |

| **Table 3. Economic evaluation lifetime results and incremental cost effectiveness ratios (ICERs)^(a)^** | | | | | | | |
| --- | --- | --- | --- | --- | --- | --- | --- |
|  | **FFIT-FU-I** | | **Hypothetical ‘no active intervention’ control scenarios** | | **ICER** | | |
|  | **Disc costs (£)**  **(95% CI)** | **Disc^(b)^ QALYs**  **(95% CI)** | **Disc costs (£)**  **(95% CI)** | **Disc QALYs**  **(95% CI)** | **Incr^(c)^ costs (£)**  **(95% CI)** | **Incr QALYs**  **(95% CI)** | **ICER cost per QALY (£)** |
| **Base Case** | 27,400  (27,200, 27,500) | 65·8  (65·8, 65·8) | 25,700  (25,600, 25,800) | 65·0  (65·0, 65·1) | 1,680  (1,480-1,870) | 0·781  (0·732-0·831) | 2,150 |
| **SA1** | 27,400  (27,200, 27,500) | 65·8  (65·8, 65·8) | 25,700  (25,600, 25,800) | 65·0  (65·0, 65·0) | 1,640  (1,460-1,840) | 0·782  (0·736-0·828) | 2,100 |
| **SA2** | 27,400  (27,300, 27,500) | 65·8  (65·8, 65·8) | 25,900  (25,800, 26,000) | 65·1  (65·1, 65·2) | 1,500  (1,330- 1,660) | 0·679  (0·648 – 0·723) | 2,200 |
| **SA3** | 27,400  (27,200, 27,500 | 65·8  (65·8, 65·8) | 25,900  (25,800, 26,000) | 65·1  (65·1, 65·1) | 1,510  (1,330 – 1,710) | 0·691  (0·648-0·736) | 2,190 |
| **SA4** | 27,400  (27,200, 27,500) | 65·8  (65·8, 65·8) | 25,900  (25,800, 26,000) | 65·0  (65·0, 65·0) | 1,450  (1,240-1,620) | 0·809  (0·761-0·851) | 1,800 |
| **SA5** | 27,400  (27,200, 27,500) | 65·8  (65·8, 65·8) | 25,900  (25,800, 26,000) | 65·0  (65·0, 65·0) | 1,470  (1,270-1,690) | 0·821  (0·770-0·876) | 1,790 |
| ^(a)^: To 3 significant figures. ^(b)^: Disc=discounted (costs and utilities were discounted at 3·5% following NICE guidance.^21^ ^(c)^: Incr=incremental. | | | | | | | |

Figure 1: Cost-effectiveness acceptability curves for the six hypothetical ‘no active intervention’ scenarios (Basecase, Scenarios 1-5 [SA1-5]), and the sensitivity analysis limiting the effect of the FFIT intervention to 5.5 years
